# Cooperative Networked PIR Detection System for Indoor Human Localization †

**DOI:** 10.3390/s21186180

**Published:** 2021-09-15

**Authors:** Chia-Ming Wu, Xuan-Ying Chen, Chih-Yu Wen, William A. Sethares

**Affiliations:** 1 Department of Electrical Engineering, National Chung Hsing University, Taichung 402, Taiwan; g107064014@mail.nchu.edu.tw (C.-M.W.); g109064007@mail.nchu.edu.tw (X.-Y.C.); 2 Department of Electrical Engineering, Bachelor Program of Electrical Engineering and Computer Science, Innovation and Development Center of Sustainable Agriculture (IDCSA), National Chung Hsing University, Taichung 402, Taiwan; 3 Department of Electrical and Computer Engineering, University of Wisconsin-Madison, Madison, WI 53706, USA; sethares@wisc.edu

**Keywords:** pyroelectric infrared sensor networks, non-wearable system, cooperative indoor localization, signal processing

## Abstract

Pyroelectric Infrared (PIR) sensors are low-cost, low-power, and highly reliable sensors that have been widely used in smart environments. Indoor localization systems can be categorized as wearable and non-wearable systems, where the latter are also known as device-free localization systems. Since the binary PIR sensor detects only the presence of a human motion in its field of view (FOV) without any other information about the actual location, utilizing the information of overlapping FOV of multiple sensors can be useful for localization. In this study, a PIR detector and sensing signal processing algorithms were designed based on the characteristics of the PIR sensor. We applied the designed PIR detector as a sensor node to create a non-wearable cooperative indoor human localization system. To improve the system performance, signal processing algorithms and refinement schemes (i.e., the Kalman filter, a Transferable Belief Model, and a TBM-based hybrid approach (TBM + Kalman filter)) were applied and compared. Experimental results indicated system stability and improved positioning accuracy, thus providing an indoor cooperative localization framework for PIR sensor networks.

## 1. Introduction

In recent years, smart environments have become a key area for Internet of Things (IoT) applications, especially in indoor localization environments (e.g., hospitals and offices). There are several localization schemes for smart system applications. For instance, for general scenarios, the system may utilize wearable devices to achieve localization. Camera-based systems may expose the private lives of individuals and infringe on privacy. Although ultrasonic sensors can provide high estimation accuracy, their narrow and directional detection field limits their use. Pyroelectric Infrared (PIR) sensors provide a useful trade-off between privacy and estimation accuracy in indoor localization systems.

The operational principle of a PIR sensor is to directly interpret the thermal variations caused by target (human) motions occurring in its field of view (FOV). The analog output of the PIR signal measures only temperature, and so does not violate privacy in the way cameras can. Various examples of PIR sensor applications can be found in the literature, focusing on the specific topic of monitoring and tracking, but the number of implemented PIR solutions is small. Moreover, although the existing studies combine the properties of PIR sensors and spatial information to estimate the target position, they lack a comprehensive analysis concerning the impact of spatial resolution on system performance and implementation complexity. Hence, the challenge of improving the performance (e.g., spatial resolution, response speed, and estimation accuracy) of the PIR sensor network remains an important problem.

Since the performance of a tracking application is closely related to the PIR detector design, our previous work [1] presented a prototype design for the PIR sensing modules and detectors consisting of two PIR detectors that integrated the sensing signals to estimate the target’s position. Considering the limitations of the existing systems, this paper reexamined the detector structure, provided improved parameter settings, and proposed signal processing algorithms and refinement strategies to improve system performance and further characterize the behaviors of PIR sensor networks. Three refinement schemes were studied: the Kalman filter [2], a Transferable Belief Model (TBM) [3], and a hybrid approach that combines a TBM with a Kalman filter. 

For the system with Kalman filter, the estimated tracking path is mainly described by the position estimates, which may not sufficiently reveal the information about the regions traversed. For the system with TBM, although it may perform no better than the Kalman filter in terms of estimation accuracy, the derived probability distribution of the target may circumvent the drawbacks of the Kalman filter and the information of target distribution can be used to calibrate the input information for the Kalman filtering. This study further develops a hybrid approach that integrates a belief-driven approach (TBM) and a model-based approach (Kalman filter). These two approaches operate together by describing the sensing status and the quantized belief with TBM, and then applying the TBM output to the estimation procedures of the Kalman filter. The tracking performances are compared and contrasted in Section 5.3.

The contributions and key features of this paper are:

(1) Distributed Tracking Framework: Based on ceiling-mounted PIR sensors, this paper provided a distributed indoor tracking framework for PIR sensor networks, extracting key features from the PIR signals for obtaining reliable information of detection zones. The proposed system with the refinement approaches was implemented in an Arduino microcontroller board, which provides real-time human tracking information through a web-based visualization interface.

(2) Estimation Refinement: A Kalman filter was applied as a quantitative benchmark to show performance improvement. For a TBM tracking scheme, this paper integrated the quantified beliefs and the weighted mean center scheme with the sensor deployment and the status of sensing zones simultaneously, which not only qualitatively provides the information about the regions traversed, but also quantitatively estimates human movements. Furthermore, due to certain limitations in modeling and considering the overall system performance, this study developed a TBM-based hybrid scheme, which was shown to be a promising extension.

The paper is organized as follows: Section 2 describes related work about PIR sensing and localization methods. Section 3 extends our preliminary design concept in [1] with several signal processing algorithms. Section 4 describes the system deployment, the signal verification, and signal processing strategies that implement the Kalman filter, TBM, and the hybrid approaches. Section 5 reports the experimental results and evaluates the system performance. Discussions are presented and suggestions are made for further research in Section 6.

## 2. Related Works

This section reviews related works on tracking human movements, focusing on measurement structure and sensing structure.

### 2.1. The Measurement Structure

Based on measurement availability, various techniques have commonly been employed for wireless indoor positioning, such as distance-based tracking systems (e.g., ranging techniques with receiver signal strength indicator, time of arrival, time difference of arrival, bearing-based tracking systems (e.g., angle of arrival), and radio frequency-based techniques (e.g., Light-emitting diode (LED) based visible light positioning networks)). For instance, Naseri [4] proposed an algorithm to solve the problem of cooperative distributed localization using joint distance and direction estimates without prior information. Zhong [5] quantitatively investigated bearing-based localization accuracy from the perspective of network geometric structure. Keskin [6] proposed a cooperative localization for hybrid infrared and visible light networks, involving multiple LED transmitters having known locations (e.g., on the ceiling) and visible light communication units equipped with both LEDs and photodetectors for cooperation. Although the above techniques achieve high accuracy in position estimation, the implementation complexity is high. For devices of limited capability, range-free localization schemes may be a sensible compromise. Mautz [7] argued that indoor positioning is more suitable for non-wearable technology since the target does not need to be equipped with any device. Since PIR sensors can have a highly plastic field of view and can convert the thermal variations of the targets motion into a sine-like waveform, they may play an important role in the development of indoor localization.

### 2.2. The Sensing Structure

Two typical methods used to detect human motion with infrared sensors are pyro-electric and thermopile.

#### 2.2.1. Thermal Sensor Array

For the thermopile method, the sensing application focuses on occupancy and human detection. Thermopiles have the potential to be low cost because they can be fabricated by CMOS processes. However, the thermoelectric methods are of low efficiency and are less sensitive than pyro-electric methods. Based on low-pixel count sensor arrays, Tyndall [8] and Walmsley-Eyre [9] proposed occupancy estimation sensor systems with machine learning classifiers for interpreting the raw data from the detector array to infer the number of occupants in the sensor’s FOV.

Savazzi [10] proposed a Bayesian tool for tracking multiple bodies through real-time analysis of thermal signatures extracted from an 8 × 8 thermopile sensor array (i.e., 64 sensors), which monitors a 2.5 m square area when mounted on a 3.0 m ceiling and tracks people with an accuracy of about 0.5 m. However, considering the system complexity and the number of sensors used, the system may be limited to certain deployment scenarios in IoT applications. Naser [11] presented a framework based on a deep convolutional encoder-decoder network to perform semantic segmentation of the human presence and estimate the occupancy in the indoor environment. However, classification and regression machine learning approaches are required to perform human segmentation and occupancy estimation.

#### 2.2.2. PIR Sensing

For the PIR sensing method, human monitoring mainly focuses on the estimation of moving trajectory, using a PIR sensor network. As depicted in [12], when several activity zones (i.e., zones of interest) are close to each other within the room, the sensors of two zones may overlap the adjacent zone(s) of interest. In this case, the FOV’s will inevitably overlap. This overlapping sensing structure can generate more zones and increase the spatial resolution, though the issue arises of how to effectively detect the target presence based on the limited sensing property of PIR sensors.

To overcome the issue of the invalid intersections caused by the deployment of overlapping FOVs, Yang proposed human tracking solutions in [13] (resp. [14]), using (resp. free form) classifiers with a training phase. Because the positioning environment is unpredictable, systems that require training are less general and harder to install and operate, even if they may have good performance in specific environments. Moreover, nine sensor nodes (PIR detectors) on the floor are used in [13,14] to enable human tracking, where each node consists of six sensors. Although these studies have acceptable estimation accuracy (about 0.4~0.5 m), such sensor nodes may be cost-prohibitive, and the deployment and time-consuming training may render them impractical.

Luo [15] proposed a scheme using ceiling-mounted infrared sensors. Four sensor modules, each with five detectors, are mounted on the ceiling of the monitored field. The FOV of each detector is modulated by a Fresnel lens array to implement the desired spatial segmentation. However, the system model is validated only by simulation. Zappi [16] demonstrated how low-power, low-cost devices can provide a rough estimate of people’s movements, correlating location with possible regions. A system using wall-mounted PIR detectors was proposed in [17] that uses TBM to combine evidence from the “prediction” and the “observation” phases which respectively use the previous and the current sensing states of the system to estimate the target trajectory. Unfortunately, the system only provides information about the regions traversed, which may make it difficult to apply in more general settings.

Brady [18] suggested that with a reference structure (also called mask), a modulation of radiation properties (e.g., absorption, permittivity, and polarization) may be applied for object analysis, including imaging, parameter estimation (e.g., location, orientation, size, velocity, or trajectory), and classification (e.g., object identity, type, or group). Therefore, FOV modulation methods can be applied to perform target tracking. Shen [19] proposed a design, considering the FOV of a PIR detector with a hemispherical Fresnel lens array in a cone-shape space, and its projection on the ground plane is an area with several concentric rings. Lu [20] illustrated the tracking experimental settings, where the experiments are conducted in a 6 m × 6 m observation space and sixteen PIR sensor nodes (32 sensors) are deployed on four walls with pegboards. The Fresnel lens arrays and coded masks were applied to subdivide the FOV of each sensor and encode the sub-regions. A distributed message passing framework is discussed with a factor graph model for target tracking. The experimental results demonstrated good tracking performance after sensor selection and calibration with a tracking resolution of 0.35 m.

**Figure 1:**
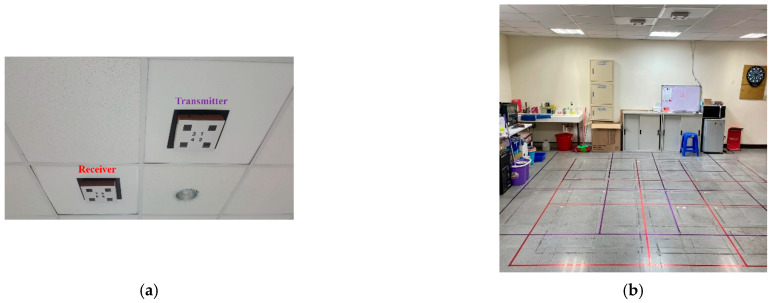
The deployment of a PIR sensor network: (**a**) a pair of PIR detectors and (**b**) the experimental scenario with grid zones.

Table 1 summarizes the characteristics of several PIR detection systems in terms of the sensor location, signal preprocessing techniques, and observation space. As shown in Figure 1 and referring to our previous work [1], this paper extended its easy-to-implement system structure, including the PIR detector design, sensing signal processing, and the deployment of the sensor network. In the experimental settings, we explored the impact of PIR detector design and signal-processing algorithms on estimation accuracy and made a comparison among the proposed refinement approaches (as detailed in Section 4 and Section 5).

## 3. Pyroelectric Infrared Detection

This section presents the design of a PIR detector and derives signal processing algorithms for extracting PIR signals.

### The Design of PIR Detector

**Figure 2:**
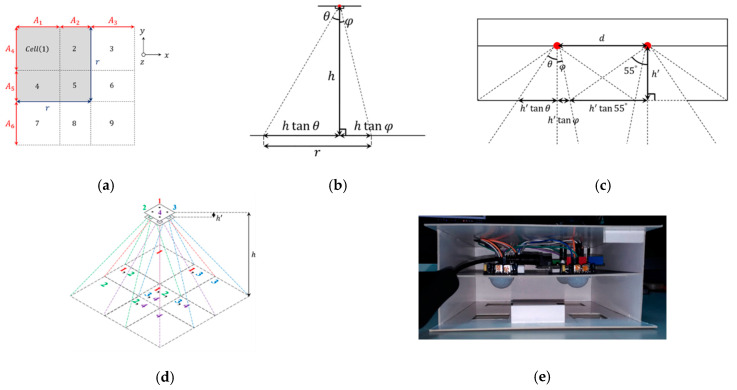
A PIR detector: (**a**) the top view of the detection area, (**b**) the detection area of a PIR module, (**c**) parameters of the reference structure, (**d**) the full view of a PIR detector, and (**e**) the inside view of the reference structure.

The PIR detectors used in this study are based on the prototype in [1] with structural improvements, especially in the parameter settings of the PIR detector. The PIR detector is mainly composed of four PIR modules, a reference structure, and a control unit, where the detection angle of a PIR module is about 110 degrees, and the maximum detection ranges are 5~7 m at the center and 3~4 m at the edge, respectively. With the above sensing characteristics, a reference structure is proposed to perform indoor localization. As shown in Figure 2a, the reference structure is used to divide the detection area of four PIR modules into nine cells, where the (square) detection area of a PIR module (gray area) covers four cells (say Cells 1, 2, 4, and 5) with side length $r=A_{1}+A_{2}=A_{4}+A_{5}$, where $A_{1}=A_{3}=A_{4}=A_{6}$ and $A_{2}=A_{5}$. Based on the above layout of the detection areas, Figure 2b shows that the detection area of a PIR module is divided into two parts by a vertical line, where θ (resp. φ) represents the degree of the left (resp. right) part, which leads to r=htanθ+htanφ. As depicted in Figure 2c, the height between a PIR module and the bottom of the reference structure is $h^{\prime }$. To reduce the overlapping detection area of the PIR modules, there is a constraint for the distance between a pair of PIR modules, $d\geq h^{\prime }\tan\varphi +h^{\prime }\tan55^{{}^{\circ}}$. The side length of the (square) holes for receiving IR radiation in the bottom is $h^{\prime }\tan\theta +h^{\prime }\tan\varphi .$ With the floor-to-ceiling height h=2.4 m, $h^{\prime }=4$ cm, d=9 cm, $h^{\prime }\tan\theta =2.7\;\mathrm{cm}$, and $h^{\prime }\tan\varphi =0.8\;\mathrm{cm}$, the size of the detection area of a PIR detector is about $3.33\times 3.33\;m^{2}$. Referring to the descriptions of d, $h^{\prime }$, $h^{\prime }\tan\theta$, and $h^{\prime }\tan\varphi$, Figure 2d illustrates the design layout and Figure 2e shows the construction of a prototype PIR detector, including an Arduino and four PIR modules.

The experimental result shows that when the target moves from a cell with the greater overlap of detection areas to a cell with a lesser degree of overlap, the PIR signals may have difficulty representing the target trajectory. This can occur when the PIR signals interfere with the detection properties of the PIR sensors, and readers may refer to [21] for an analysis of their dynamic behavior.

To effectively determine whether a PIR sensor is triggered, a signal normalization method (e.g., the short-time energy (STE) method [22]) may be performed, which normalizes the analog PIR signals (voltages) to more clearly indicate the state of each PIR sensor. We refer the reader to [1] for more details. With a single PIR detector, the matrix of N-times sampled normalized PIR signals $S_{PIR}$ is

(1) $$S_{PIR}={\left[s_{n,p}\right]}_{N\times P}={\left[s_{n}\right]}_{N\times 1},$$

where n=1,…,N, p=1,…,P, $s_{n,p}$ is the state of the *p*th PIR sensor in the nth sampling (i.e., $s_{n,p}=0$ (*resp*. 1), indicating the pth PIR sensor is *not triggered* (*resp. triggered*)), the nth normalized PIR signal $s_{n}=\left[\begin{array}{ccc} s_{n,1} & \cdots & s_{n,P} \end{array}\right]$ is a 1×P row vector, and P is the number of the PIR sensors (modules) in a detector. A C×P matrix, Cov*_det_*, built to describe each cell covered by the detection area(s) of which PIR module(s) in a detector, is represented as

(2) $${\mathrm{Cov}}_{det}={\left[{\mathrm{cov}}_{c,p}\right]}_{C\times P}={\left[{\mathrm{cov}}_{c}\right]}_{C\times 1},\;\mathrm{with}\;c=1,\ldots ,C$$

where c is the cell index, C is the number of cells of the detection area of a detector, ${\mathrm{cov}}_{c,p}$ indicates the detection area of the pth PIR module which covers Cell(c) (i.e., ${\mathrm{cov}}_{c,p}=0$ (*resp.* 1) indicates Cell(c) is *not covered* (*resp. covered*)), and ${\mathrm{cov}}_{c}=\left[\begin{array}{ccc} {\mathrm{cov}}_{c,1} & \cdots & {\mathrm{cov}}_{c,P} \end{array}\right]$ is a 1×P row vector. 

Taking a PIR detector with P=4 and C=9 in this work, ${\mathrm{Cov}}_{det}$ can be represented as follows:

(3) $$\begin{array}{l} {\mathrm{Cov}}_{det}={\left[\begin{array}{ccc} {\mathrm{cov}}_{1,1} & \cdots & {\mathrm{cov}}_{1,4}\\ \vdots & \ddots & \vdots \\ {\mathrm{cov}}_{9,1} & \cdots & {\mathrm{cov}}_{9,4} \end{array}\right]}_{9\times 4}={\left[{\mathrm{cov}}_{c}\right]}_{9\times 1},\\ \;=\left[\begin{array}{c} \left[\begin{array}{cccc} 1 & 0 & 0 & 0 \end{array}\right]\\ \vdots \\ \left[\begin{array}{cccc} 0 & 0 & 0 & 1 \end{array}\right] \end{array}\right]. \end{array}$$

Conceptually, we can rely on whether “$s_{n}$ is zero (null) vector 0” or “$s_{n}\in {\mathrm{Cov}}_{det}$” to determine the presence of the target in the detection area of a PIR detector. However, in practice, $s_{n}$ usually does not correspond exactly to 0 when $s_{n}\notin {\mathrm{Cov}}_{det}$. On the other hand, when $s_{n}\in {\mathrm{Cov}}_{det}$, the cell displayed by $s_{n}$ may be far away from the true location of the target. This occurs because the STE uses analog PIR signals which are easily affected by the gait and posture of the target along with other environmental factors. Therefore, even though the STE is a good way to determine the state of each PIR sensor, normalized PIR signals still cannot provide explicit feedback on which cell the target activates.

**Figure 3:**
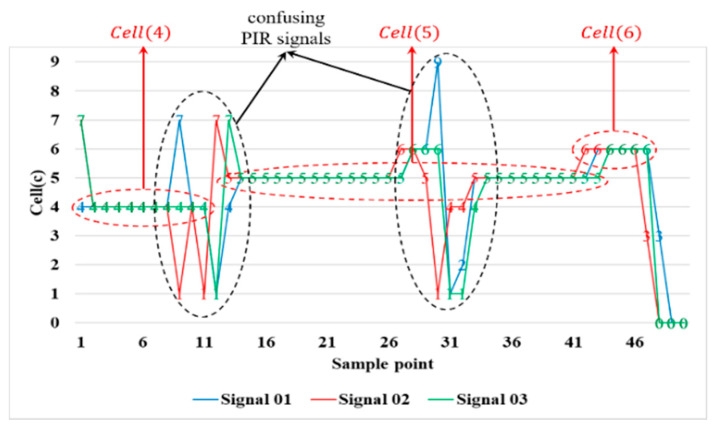
Feature PIR signals: moving from *Cell*(4) to *Cell*(6).

To this effect, the “feature PIR signal” is extracted once the number of $s_{n}$ reaches $N^{\prime }$ (i.e., containing $N^{\prime }$ normalized PIR signals). Denote the $N^{\prime }$ normalized PIR signals as a frame, ${\left[s_{n}\right]}_{N^{\prime }\times 1}$ with *n* = 1, …, $N^{\prime }$. The PIR signal feature $s_{feat}$ is the signal type that most frequently occurs in the $N^{\prime }$-times sampled signals, which has to be either $s_{feat}=0$ or $s_{feat}\in {\mathrm{Cov}}_{det}$. For instance, $s_{feat}=\left[\begin{array}{cccc} 1 & 1 & 1 & 1 \end{array}\right]={\mathrm{cov}}_{5},$ suggesting that the target is in Cell(5). Figure 3 shows the process of the target movements from Cell(4) to Cell(6) and the corresponding feature PIR signals measured by the PIR detector.

In our previous work [1], the feature signal extraction was not performed, which may have led to inaccurate or false measurement points. As shown in Figure 3, when the target moved between two adjacent cells, it generated confusing PIR signals. For instance, consider the target’s movement from Cell(5) with a higher degree of overlap to Cell(6) (with a lower degree of overlap). The PIR signal still indicates that the target was in Cell(5) after receiving these confusing PIR signals. Therefore, to facilitate decision-making about whether the target leaves Cell(5) heading towards Cell(6), a cooperative scheme was applied (i.e., two PIR detectors work cooperatively, having an overlapping detection area with its adjacent PIR detector) to obtain improved target detectability. The following section details the operations with two PIR detectors.

## 4. Indoor Human Localization System

To improve the spatial resolution, this section utilizes the sensing data to estimate the target position and the possible region of the target with two PIR detectors operating cooperatively. To verify and refine the system performance, Kalman filter, TBM, and TBM-based approaches are described and implemented.

### 4.1. The Deployment of the Detection Area

**Figure 4:**
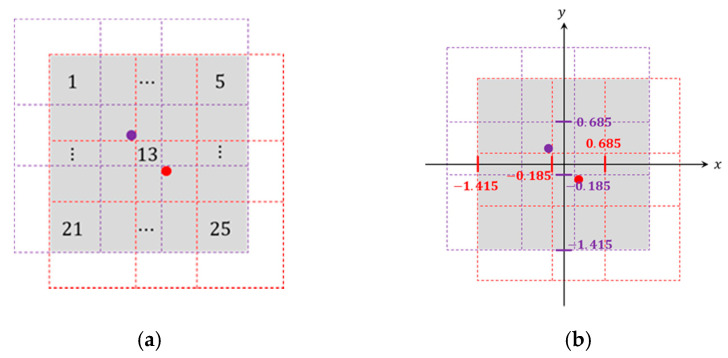
(**a**) The detection area of the proposed system, where the red (resp. purple) point is Rec (resp. Trans1) and (**b**) the two-dimensional coordination of Rec (resp. Trans1) is (0.25, −0.25) (resp. (−0.25, 0.25)).

Let a receiver (Rec) and a transmitter (Trans1) denote the two PIR detectors, where the detection area of the system (gray area) is the overlapping detection regions of these two detectors. Note that Zone(⬝) represents the overlap of two cells from one of the cells of Rec and Trans1, which is covered by the detection area of the PIR modules. Denote $\left(cx_{z},cy_{z}\right)$ as the central coordination of Zone(z) with z=1,…,Z, where z is the zone index, Z is the total number of zones. Referring to Figure 4, the detection system is with Z = 25 and $\left(cx_{13},cy_{13}\right)=\left(0,0\right)$. According to the PIR signal processing scheme in Section 3, a single-sample feature PIR signal is composed of the state of each PIR sensor. Alternatively, we can also transform the type of feature PIR signals using the central coordination of Zone(z).

### 4.2. The Operation of the System

Based on the system deployment of the detection area and the PIR signals extraction method of Section 3, the general expression of the zone coverage yields

(4) $$\begin{array}{l} {\mathrm{Cov}}_{sys}={\left[\begin{array}{c} {}_{d}{\mathrm{cov}}_{z,p} \end{array}\right]}_{Z\times \left(D\times P\right)},\\ \;=\left[\begin{array}{ccc} \left[\begin{array}{ccc} {}_{1}{\mathrm{cov}}_{1,1} & \cdots & {}_{1}{\mathrm{cov}}_{1,P} \end{array}\right] & \cdots & \left[\begin{array}{ccc} {}_{D}{\mathrm{cov}}_{1,1} & \cdots & {}_{D}{\mathrm{cov}}_{1,P} \end{array}\right]\\ \vdots & \ddots & \vdots \\ \left[\begin{array}{ccc} {}_{1}{\mathrm{cov}}_{Z,1} & \cdots & {}_{1}{\mathrm{cov}}_{Z,P} \end{array}\right] & \cdots & \left[\begin{array}{ccc} {}_{D}{\mathrm{cov}}_{Z,1} & \cdots & {}_{D}{\mathrm{cov}}_{Z,P} \end{array}\right] \end{array}\right],\\ =\left[\begin{array}{ccc} {}_{1}{\mathrm{cov}}_{1} & \cdots & {}_{D}{\mathrm{cov}}_{1}\\ \vdots & \ddots & \vdots \\ {}_{1}{\mathrm{cov}}_{Z} & \cdots & {}_{D}{\mathrm{cov}}_{Z} \end{array}\right]={\left[\begin{array}{c} {\mathrm{cov}}_{z} \end{array}\right]}_{Z\times 1}, \end{array}$$

where $d=1,\ldots ,D,\;{}_{d}{\mathrm{cov}}_{z,p}$ indicates the detection area of the pth PIR module in the dth detector which covers Zone(z). Note that ${}_{d}{\mathrm{cov}}_{z,p}=0$ (*reap.* 1) indicates Zone(z) is *not covered* (*reap. covered*)), $\begin{array}{c} {\mathrm{cov}}_{z} \end{array}=\left[\begin{array}{ccc} {}_{1}{\mathrm{cov}}_{z} & \cdots & {}_{D}{\mathrm{cov}}_{z} \end{array}\right]$ is a 1×D row vector with D=2 in this work.

To improve the localization accuracy and describe the tendency of a moving target, the Kalman filter (*resp*. TBM model) was applied to obtain detailed information about a target trajectory with the input $\left(cx_{z},cy_{z}\right)$ (*resp.* the state of each PIR sensor $s_{k}$, corresponding to the zones concerning the target movement). To obtain applicable feature PIR signals for the TBM model, a threshold thr was applied to indicate the minimum number of occurrences of the signal type $s_{k}$ (e.g., when $s_{k}={\left[\begin{array}{c} 1 \end{array}\right]}_{1\times \left(D\times P\right)}$, the number of times of the signal type ${\left[\begin{array}{c} 1 \end{array}\right]}_{1\times \left(D\times P\right)}$ reaches thr in the feature PIR signals). Note that thr may be set according to the actual environment. In our experiments, the value of thr was set to four. The algorithm is summarized in Algorithm 1.

**Algorithm 1:** The Inputs of TBMDenote ${}^{m}s_{feat}$ as the feature PIR signal of ***m***th frame. Denote $s_{k}$ as the ***k***th input of TBM. ► **Initialization:** 1: *k* ← 1 2: *Z* ← 25 3: *thr* ← 4 4: $Count\leftarrow {\left[0\right]}_{\left(Z+1\right)\times 1}$  ▷ Record the number of times of each signal type appearing in the *m*th frame. 5: ${Count}^{\prime }\leftarrow {\left[0\right]}_{Z\times 1}$  ▷ Record the number of observations of each signal type 6: ${\mathrm{Cov}}_{sys}\leftarrow {\left[{\mathrm{cov}}_{z}\right]}_{Z\times 1}$  ▷ It is used for signal type comparison. ► **Start:**      ➤ *Get s_k_* 7:    **if**
${}^{m}s_{feat}\neq 0$ **then** 8:    ${Count}^{\prime }\left(index_{max}\right)\leftarrow {Count}^{\prime }\left(index_{max}\right)+1$ 9:     **for**
*z* ← 1 **to**
*Z*
**do** 10:     **if**
${Count}^{\prime }\left(z\right)=thr$ **then** 11:      **if**
${\mathrm{Cov}}_{sys}\left(z\right)\neq s_{k-1}$ **then** 12:       $s_{k}\leftarrow {\mathrm{Cov}}_{sys}\left(z\right)$ 13:       k←k+1      ➤ *Resetting* 14:       ${Count}^{\prime }\leftarrow {\left[0\right]}_{Z\times 1}$ 15:       **break** 16:      **else** 17:       ${Count}^{\prime }\leftarrow {\left[0\right]}_{Z\times 1}$ 18:       **break** 19:      **end if** 20:     **end if** 21:    **end for** 22:   **end if** 23: $Count\leftarrow {\left[0\right]}_{\left(Z+1\right)\times 1}$

### 4.3. Transferable Belief Model (TBM)

The TBM is a theoretical framework that can be used to describe quantified beliefs about a set of hypotheses, and the description of belief is carried out at two levels: (1) a credal level—using belief functions to quantify beliefs and (2) a pignistic level—using beliefs to make decisions with probability functions. Instead of mainly using the TBM to describe the target trajectory as shown in [17], we propose an integrated TBM method, considering the deployment and the status of sensing zones simultaneously.

Firstly, define the frame of discernment (FoD) for the movement prediction at each zone, which yields

(5) $$\Omega_{pre}=\left\{Inside,\;\bar{Inside}\right\},$$

and the power set of $\Omega_{pre}$ is

(6) $$2^{\Omega_{pre}}=\left\{\emptyset ,Inside,\bar{Inside},\Omega_{pre}\right\}.$$

Similarly, with the FoD of observation,

(7) $$\Omega_{obs}=\left\{Inside,\;\bar{Inside}\right\},$$

and the power set of $\Omega_{obs}$ is

(8) $$2^{\Omega_{obs}}=\left\{\emptyset ,Inside,\bar{Inside},\Omega_{obs}\right\},$$

where Inside (*resp.* $\bar{Inside}$) represents the hypothesis that the target *is* (*resp. is not*) in Zone(z). Since the proposed system explicitly indicates the presence of a target, no beliefs are given to contradictory and unreasonable hypotheses (e.g., ∅, $\Omega_{pre}$ and $\Omega_{obs}$). Accordingly, the sets of focal elements of prediction and observation are as follows:

(9) $$\xi \Omega_{pre}=\left\{Inside,\;\bar{Inside}\right\},$$

(10) $$\xi \Omega_{obs}=\left\{Inside,\;\bar{Inside}\right\}.$$

Next, we explain the calculation process of the TBM and model settings from credal and pignistic levels.

#### 4.3.1. Credal Level

*(1) Basic Belief Assignment*: To quantify the elements H of the power set $2^{\Omega }$ (i.e., $H\in 2^{\Omega }$), define the mass function m, which satisfies

(11) $$\sum_{H\in 2^{\Omega }}m\left(H\right)=1\;\mathrm{and}\;m\left(H\right)\in \left[0,1\right].$$

The following are the mass functions, defined for two independent evidence sources: prediction and observation.

*Prediction Mass Function*: the mass function of prediction in Zone(z), z=1,…,Z at the kth sample is

(12) $$\begin{array}{ll} m_{pre}^{z}\left(Inside,k\right)= & \gamma_{stay}\cdot {\hat{P}}_{k-1}^{z}\left(Inside\right)\\ & +\gamma_{move}\cdot \sum_{u=1}^{N_{u}}{\hat{P}}_{k-1}^{u}\left(Inside\right)\\ & +\gamma_{jump}\cdot \sum_{w=1}^{N_{w}}{\hat{P}}_{k-1}^{w}\left(Inside\right), \end{array}$$

(13) $$m_{pre}^{z}\left(\bar{Inside},k\right)=1-m_{pre}^{z}\left(Inside,k\right),$$

where $N_{u}$ (*resp.* $N_{w}$) is the number of neighboring (*resp.* far) zones of Zone(z); ${\hat{P}}_{k-1}^{z}\left(Inside\right)$, ${\hat{P}}_{k-1}^{u}\left(Inside\right),$ and ${\hat{P}}_{k-1}^{w}\left(Inside\right)$ are, respectively, the normalized target presence probability in Zone(z), Zone(u), and Zone(w); $\gamma_{stay}$, $\gamma_{move},$ and $\gamma_{jump}$ are parameters of the human motion model, which respectively represent the probability of staying in the same zone, moving to a neighbor zone, and jumping to a far zone. Readers may refer to [23] for parameter settings.

*Observation Mass Function*: the mass function of observation in Zone(z) at the kth sample is as follows:

(14) $$m_{obs}^{z}\left(Inside,k\right)=\left[\frac{N_{Tri}^{z}\left(k\right)}{N_{Tot\_tri}\left(k\right)}\right]\cdot \left[\frac{N_{Tri}^{z}\left(k\right)}{N_{\mathrm{Cov}}^{z}}\right],$$

(15) $$m_{obs}^{z}\left(\bar{Inside},k\right)=1-m_{obs}^{z}\left(Inside,k\right),$$

where $N_{Tri}^{z}\left(k\right)$ represents the number of triggered sensors in Zone(z); $N_{Tot\_tri}\left(k\right)$ is the total number of triggered sensors in the whole network; $N_{\mathrm{Cov}}^{z}=N_{Tri}^{z}\left(k\right)+N_{\bar{Tri}}^{z}\left(k\right)$ is the number of sensors covering Zone(z); and $N_{\bar{Tri}}^{z}\left(k\right)$ represents the number of non-activated sensors in Zone(z).

*(2) Combination Rule of Dempster–Shafer (D-S) Evidence Theory (D-S Rule)*: Combining the mass functions of $n_{e}$ independent evidence sources $m_{1},\ldots ,m_{n_{e}}$ into a single function $m_{1⨁\ldots ⨁n_{e}}$, the conjunctive rule is then

(16) $$m_{1\cap \ldots \cap n_{e}}\left(A\right)=\sum_{A_{1}\cap \ldots \cap A_{n_{e}}=A}m_{1}\left(A_{1}\right)\cdot \ldots \cdot m_{n_{e}}\left(A_{n_{e}}\right),$$

for all $A,A_{1},\ldots A_{n_{e}}\in 2^{\Omega }$. Then, the normalized conjunctive rule (D-S rule) is

(17) $$m_{1⨁\ldots ⨁n_{e}}\left(\emptyset \right)=0,$$

(18) $$m_{1⨁\ldots ⨁n_{e}}\left(A\right)=\frac{1}{1-K}m_{1\cap \ldots \cap n_{e}}\left(A\right),$$

for all $A\in 2^{\Omega }\setminus \emptyset$, where

$$K=\sum_{A_{1}\cap \ldots \cap A_{n_{e}}=\emptyset }m_{1}\left(A_{1}\right)\cdot \ldots \cdot m_{n_{e}}\left(A_{n_{e}}\right)=m_{1\cap \ldots \cap n_{e}}\left(\emptyset \right).$$

The following is a detailed description of our case. Note the FoDs in our case were equal (i.e., $\Omega_{pre}=\Omega_{obs}=\Omega^{\prime }$).

(19) $$m_{pre⨁obs}^{z}\left(\emptyset ,k\right)=0,$$

(20) $$\begin{array}{l} m_{pre⨁obs}^{z}\left(Inside,k\right)\\ \;=\frac{1}{1-K}\left[m_{pre}^{z}\left(Inside,k\right)\cdot m_{obs}^{z}\left(Inside,k\right)\right], \end{array}$$

(21) $$\begin{array}{l} m_{pre⨁obs}^{z}\left(\bar{Inside},k\right)\\ \;=\frac{1}{1-K}\left[m_{pre}^{z}\left(\bar{Inside},k\right)\cdot m_{obs}^{z}\left(\bar{Inside},k\right)\right], \end{array}$$

(22) $$m_{pre⨁obs}^{z}\left(\Omega^{\prime },k\right)=0,$$

where

$$\begin{array}{cc} K & =m_{pre\cap obs}^{z}\left(\emptyset ,k\right)\\ & =\left[m_{pre}^{z}\left(Inside,k\right)\cdot m_{obs}^{z}\left(\bar{Inside},k\right)\right]+\\ & \;\left[m_{pre}^{z}\left(\bar{Inside},k\right)\cdot m_{obs}^{z}\left(Inside,k\right)\right]. \end{array}$$

#### 4.3.2. Pignistic Level

In a pignistic level, the probability BetP is used to transform the belief function into the probabilities of singletons x by combining the mass functions of $n_{e}$ independent evidence sources $m_{1⨁\ldots ⨁n_{e}}=m^{\prime }$, which is

(23) $$BetP\left(x\right)=\sum_{A\subseteq \Omega }\frac{m^{\prime }\left(A\right)}{\left|A\right|},\;\forall \in \Omega ,$$

where |A| is the number of elements in A. Therefore, the probability of the target in Zone(z) at the kth sampling is

(24) $$\begin{array}{ll} P_{k}^{z}\left(Inside\right) & =BetP_{k}^{z}\left(Inside\right)\\ & =m_{pre⨁obs}^{z}\left(Inside,k\right)+\frac{m_{pre⨁obs}^{z}\left(\Omega^{\prime },k\right)}{2},\\ & =m_{pre⨁obs}^{z}\left(Inside,k\right). \end{array}$$

To get the probability distribution of the target in each zone, normalize $P_{k}^{z}\left(Inside\right)$ by

(25) $${\hat{P}}_{k}^{z}\left(Inside\right)=\frac{P_{k}^{z}\left(Inside\right)}{\sum_{z=1}^{Z}P_{k}^{z}\left(Inside\right)},\;z=1,\ldots ,Z.$$

Based on the above analysis, the probability variation of the target being in each zone from the k−1th sampling to the kth sampling, $\Delta Pro_{k-1∼k}^{z}$, can be derived for describing the target movement. Typical examples and qualitative data analysis are depicted in Section 5.3.1.

#### 4.3.3. Accuracy Performance

Referring to the probability variation of the target being in each zone from the k−1th sampling to the kth sampling, $\Delta Pro_{k-1∼k}^{z}$, and the central coordination for Zone(z) (i.e., $\left(cx_{z},cy_{z}\right)$), the weighted mean center is given as

(26) $$c{\bar{x}}_{\omega }=\frac{\sum_{z=1}^{Z}cx_{z}\cdot \omega_{k}^{z}}{\sum_{z=1}^{Z}\omega_{k}^{z}},\;c{\bar{y}}_{\omega }=\frac{\sum_{z=1}^{Z}cy_{z}\cdot \omega_{k}^{z}}{\sum_{z=1}^{Z}\omega_{k}^{z}}$$

with

$$\omega_{k}^{z}=\{\begin{array}{rr} \Delta Pro_{k-1∼k}^{z}, & \Delta Pro_{k-1∼k}^{z}\geq 0\\ 0, & \Delta Pro_{k-1∼k}^{z}<0 \end{array},$$

which can be applied to represent the estimated cartesian coordinate of the target. Quantitative data analysis of the TBM method is presented in Section 5.3.2.

### 4.4. Kalman Filter

The Kalman filter [2] has been proposed for solving the discrete-data linear filter problem, which can estimate the state of a dynamical system from a series of measurements. It estimates the posteriori state at time k with the posteriori state estimate at time k−1 and the measurement at time k. Let ${\hat{s}}_{k|k}$ be the posteriori state estimate at time k with a (n×1) vector space and denote $P_{k|k}$ as the covariance matrix of estimate error of ${\hat{s}}_{k|k}$ with a (n×n) dimensional space, which is

(27) $$P_{k|k}=E\left[\left(s_{t,k}-{\hat{s}}_{k|k}\right){\left(s_{t,k}-{\hat{s}}_{k|k}\right)}^{T}\right]=E\left[e_{k|k}e_{k|k}{}^{T}\right]$$

where $s_{t,k}$ is the unknown true state at time k with a (n×1) vector space and $e_{k|k}$ is the estimated error of ${\hat{s}}_{k|k}$. The state estimate at time k (output) can be presented by two phases: prediction (time update) and correction (measurement update).

#### 4.4.1. Prediction (Time Update)

The prior state estimate at time k is ${\hat{s}}_{k|k-1}$, which yields

(28) $${\hat{s}}_{k|k-1}=\Phi_{k}{\hat{s}}_{k-1|k-1},$$

where $\Phi_{k}$ is the state-transition matrix. The covariance matrix of estimate error of ${\hat{s}}_{k|k-1}$ is

(29) $$P_{k|k-1}=\Phi_{k}P_{k-1|k-1}\Phi_{k}{}^{T}+Q_{k},$$

where $Q_{k}$ is the covariance matrix of process noise. 

#### 4.4.2. Correction (Measurement Update)

The Kalman gain is $K_{k}$, which yields

(30) $$K_{k}=P_{k|k-1}H_{k}{}^{T}{\left(H_{k}P_{k|k-1}H_{k}{}^{T}+R_{k}\right)}^{-1},$$

where $H_{k}$ is the measurement matrix. Thus, the posteriori state estimate at time k is

(31) $${\hat{s}}_{k|k}={\hat{s}}_{k|k-1}+K_{k}\left(z_{k}-H_{k}{\hat{s}}_{k|k-1}\right),$$

where $z_{k}$ is the measurement at time k. Accordingly, the covariance matrix of estimate error of ${\hat{s}}_{k|k}$ is given by

(32) $$P_{k|k}=\left(I-K_{k}H_{k}\right)P_{k|k-1},$$

where I is the identity matrix.

### 4.5. Implementation of the Localization System

To effectively utilize the computing capabilities of the microcontroller unit (MCU), based on the system operation described in Section 4.2, the sensing inputs were used to estimate the target’s trajectory. All of the processing was performed by two Arduino nodes. One is DFRobot Bluno [24], which integrates the ATmega328 microcontroller, a TI CC2540 Bluetooth 4.0 chip, and an Arduino UNO development board. The other is Bluno Mega 2560 [25], which integrates an ATmega2560 microcontroller, a TI CC2540 Bluetooth 4.0 chip, and Arduino-compatible hardware. A Bluno and a Bluno Mega 2560 respectively play the role of a transmitter and a receiver. Note that the Bluno Mega 2560 receives the sensing data from the Bluno, detects the PIRs’ signal, and runs the three algorithms (i.e., Kalman Filter, TBM, and Hybrid). Arduino microcontroller boards provide real-time tracking information through a web-based visualization interface, showing the cartesian coordinates of the target, $\left(x_{m}^{k},y_{m}^{k}\right)$.

**Figure 5:**
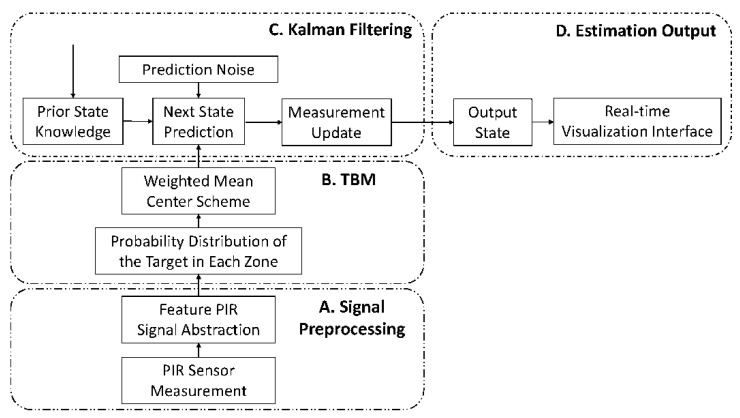
The flowchart of the proposed system with the refinement approaches.

Figure 5 shows a flowchart of the proposed refinement schemes, including Kalman filter, TBM, and TBM-based hybrid approaches. Note that the system with Kalman filter consists of parts A, C, and D; the system with TBM consists of parts A, B, and D; and the system with the TBM-based hybrid approach consists of parts A, B, C, and D. The analysis and experimental results of the refinement approaches are detailed in Section 5.3.

## 5. Experimental Results

This section evaluates the system performance and depicts the system outputs (i.e., $\left(x_{m}^{k},y_{m}^{k}\right)$ and $\Delta Pro_{k-1∼k}^{z}$), obtained by refining the feature PIR signals. Three experiments were conducted to assess the system feasibility. Experiment 1 validated the system parameters. Experiment 2 explored the qualitative performance of TBM tracking. Experiment 3 quantitatively analyzed the Kalman filter, TBM, and the TBM-based hybrid approaches.

Figure 1b illustrates the experimental scenario with grid zones of the proposed system in a 5 m × 5 m observation space. The PIR signals are described in Section 3 and Section 4.3, and Figure 4a shows the selected routes as a reference, including:

- Route 01: slash walking from the bottom left corner to the top right corner, e.g., Zone(21→17→13→9→5) or Cell(3→5→7);
- Route 01*: slash walking from the top left corner to the bottom right corner, e.g., Zone(1→7→13→19→25);
- Route 02: walking along horizontal zones, e.g., Zone(16→17→18→19→20) or Cell(4→5→6);
- Route 03: walking along the boundary line between two neighboring zones, e.g., Zone(4,5→9,10→14,15→19,20→24,25);
- Route 04: walking along a V-shaped route from the top right corner to the bottom center to the top left corner, e.g., Zone(5→9→14→19→23→17→12→7→1);
- Route 05: walking along a square route in a clockwise direction, e.g., Zone(5→10→15→20→25→24→23→22→21→16→11→6→1→2→3→4→5).

The numbers of repetitions of the tests for Route 1*, Route 1, Route 2, Route 3, Route 4, and Route 5 were 50, 50, 50, 10, 10, and 10, respectively. The tests were conducted by an average-size man (average medium frame being 5’6” with a weight of 145 pounds). The impacts of different types of routes on system performance were examined in the following experiments, which provide a basis for the design of indoor localization applications.

### 5.1. Experiment 1: Validation of Parameters

**Figure 6:**
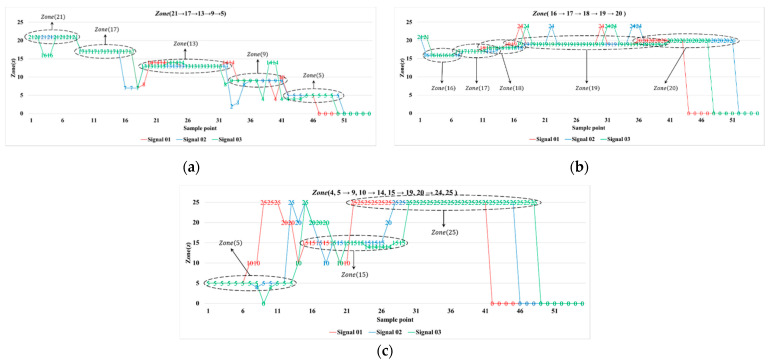
Feature PIR signals: (**a**) Route 01, (**b**) Route 02 and (**c**) Route 03.

The first set of experiment was conducted to validate the system parameters. Figure 6 illustrates typical detection scenarios of the system, operated by two PIR detectors (Rec and Trans1). Observe that with the cooperation between PIR detectors, these feature PIR signals can more clearly feedback the target trajectory.

As depicted in Figure 3 and Figure 6, both the single PIR detector and the system acting cooperatively (i.e., with two or more PIR detectors) can indicate the target trajectory. In the experimental routes in Figure 6a,b, the proportions of the applicable feature PIR signals were about 70% and 75%, respectively, which suggests that the proposed system has applicable feature PIR signals for the localization task. Although the experimental route in Figure 6c traverses two zones, the system can still effectively determine one of these two zones (about 75%). Thus, the detection performance depends on a well-designed PIR detector and the proper processing of the analog PIR signals.

### 5.2. Experiment 2: Qualitative Analysis of TBM Tracking

Experiment 2 investigated the characteristics of the TBM tracking scheme via qualitative data analysis. Corresponding to $s_{k}$ (i.e., the input to TBM), let ${\bar{\mathrm{cov}}}_{z}$ describe the zone coverage of Zone(z), which is not on the route of the target. Let ${\underline{\mathrm{cov}}}_{z}$ represent the zone coverage of Zone(z) along the route which fails to respond appropriately to the correct input sequence due to missing or redundant input(s). Table 2 shows the inputs to the TBM, obtained via the processing of Algorithm 1 with the feature PIR signals. Ideally, since each route crosses five zones, there are five inputs of TBM (i.e., the *i*th set of the TBM inputs, Input *i*, is composed of $s_{1∼5}$).

**Figure 7:**
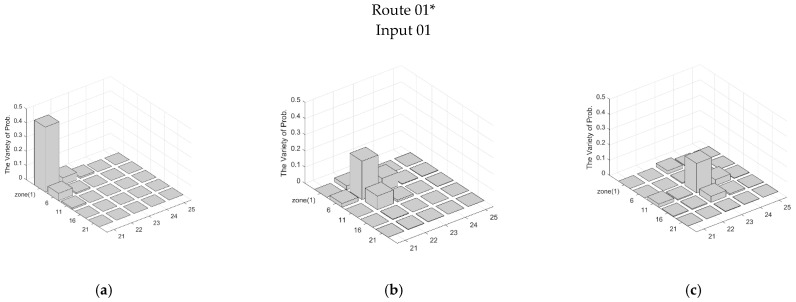

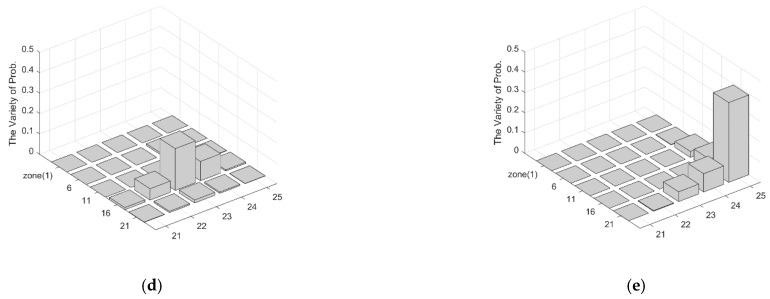
The distribution of $\Delta Prob_{k-1∼k}^{z}$ of Input 01 about Route 01*: (**a**) 0∼1st sampling interval, (**b**) 1∼2nd sampling interval, (**c**) 2∼3rd sampling interval, (**d**) 3∼4th sampling interval, and (**e**) 4∼5th sampling interval.

**Figure 8:**
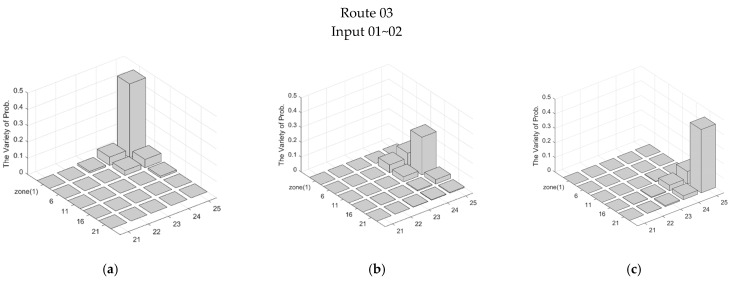
The distribution of $\Delta Prob_{k-1∼k}^{z}$ of Input 01~02 for Route 03: (**a**) 0∼1st sampling interval, (**b**) 1∼2nd sampling interval and (**c**) 2∼3rd sampling interval.

Observe that although some sets of inputs in Table 2 do not completely and correctly describe the target trajectory, these inputs still provide clues about the target trajectory. Accordingly, the derived $\Delta Prob_{k-1∼k}^{z}$ defines the possible range of the target which is composed of several zones with a variety of probabilities from the (*k*−1)~*k*th sampling interval. Note that $\Delta Prob_{k-1∼k}^{z}$ (*resp.* ${\bar{\Delta Prob}}_{k-1∼k}^{z}$) means the Zone(z) is (*resp.* is not) on the route of the target, and ${\underline{\Delta Prob}}_{k-1∼k}^{z}$ means that the Zone(z) is on the route of the target without corresponding to the correct input sequence. Considering the interference of the PIR sensors with neighbors, the top three most varied zones (i.e., the highest three $\Delta Prob_{k-1∼k}^{z}$ values) were chosen to represent the tracking tendency. Figure 7 and Figure 8 demonstrate $\Delta Prob_{k-1∼k}^{z}$ with part of inputs about Routes 01* and 03, and Table 3 presents the top three most varied zones during the (*k* − 1)~*k*th sampling interval.

Referring to Table 2, Zone(18) is not on Route 01*. However, the higher overlap degree of Zone(18), compared with Zone(19), leads to a biased input. Therefore, instead of only determining the passing zones from the inputs (i.e., the content of $s_{4}$ indicates that the target is in Zone(18)), the TBM reasonably and objectively suggests that the target initiates the sensing and detection operation in Zone(18) and Zone(19) during movement. Notice that in Table 2, Input 03 of Route 01* and Inputs 01~03 of Route 02 completely and correctly indicate the target trajectory. The following discussion focuses on the performance with biased, missing, or redundant inputs. For Route 01*, $s_{4}$ of Input 01 and Input 02 are biased, but the $s_{5}$ and $s_{6}$ of Input 02 indicate that the target is currently in Zone(19) and Zone(25), which implies that the $s_{4}$ may be a redundant input. With Inputs 01~02 of Route 03, Figure 8 shows the distributions of $\Delta Prob_{k-1∼k}^{z}$ along Route 03. Observe that although there are missing inputs, the whole distribution still reflects the target trajectory.

### 5.3. Experiment 3: Quantitative Analysis

This set of experiments examined the tracking performance of the three approaches via quantitative data analysis.

#### 5.3.1. Kalman Filter Tracking

As shown in Figure 5, the Kalman filter tracking is composed of parts A, C, and D. In the three experimental routes, the system performance (estimation accuracy) was assessed via the root mean square error (RMSE) of the two-dimensional location estimate. Since the target conducts multiple passes on each type of route, the walking posture of the target may be different each time (e.g., speed, pace, and body swing, etc.). Accordingly, to explore the estimation stability and analyze the system performance, an objective starting point and an ending point were specified for each route. The starting and ending points of Routes 01~05 (Section 4.2) were:

➢ Route 01: (−1.05,−1.05)⟶(1.415,1.415);
➢ Route 02: (−1.05,−0.435)⟶(1.415,−0.435);
➢ Route 03: (0.685,1.05)⟶(0.685,−1.415).
➢ Route 04: (0.868,1.05)⟶(0,−0.868)⟶(−0.868,1.05);
➢ Route 05: (0.868,1.05)⟶(0.868,−0.868)⟶(−0.868,−0.868)⟶(−0.868,1.05)⟶(0.868,1.05).

**Figure 9:**
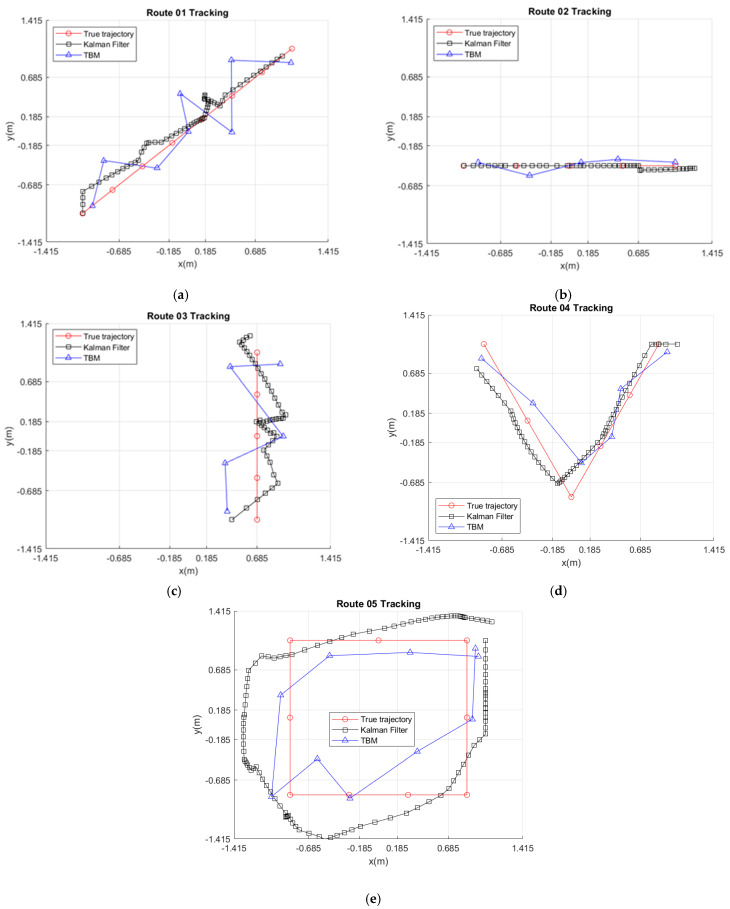
Typical examples of tracking results with Kalman filter and TBM: (**a**) Route 01, (**b**) Route 02, (**c**) Route 03, (**d**) Route 04, and (**e**) Route 05.

With the implementation in Section 4.4, Figure 9 shows the tracking performances of Routes 01~05 with the sampling time-interval 0.08 s and the noise variance 0.01.

Table 4 shows the estimation performance of Routes 01~05 under the RMSE error criterion. For Route 01, crossing the sensing field diagonally, the average RMSE relationship between *x* and *y* is approximately linear with a slope of 1. For Route 02, walking along horizontal zones, the dominant source of estimation error is from the *x*-direction movement. For Route 03, walking along the boundary line between two adjacent zones in the vertical direction, the average RMSE relationship between *x* and *y* is approximately linear with a slope of 2/3, which implies that the dominant source of estimation error is from the *y*-direction movement. For Route 04, similar to the diagonal route of Route 01, a V-shaped route makes the average RMSE relationship between *x* and *y* approximately linear with a slope of 1. However, for Route 05, the Kalman filter tracking had significant performance degradation, due to a relatively slow response concerning the changes in direction.

#### 5.3.2. TBM Tracking

Referring to Figure 5 (including Parts A, B, and D), Figure 9 illustrates the estimated target trajectory via the TBM method and the weighted mean center scheme. Compared with the Kalman filter tracking, the TBM method generates a relatively high-frequency zigzag shape. Instead of indicating the possible range of the target, as depicted in the qualitative analysis, the correctness of the input sequence and the weighted mean center scheme provide a way to describe the target trajectory. Table 5 shows the tracking performance of Routes 01~05 under the RMSE error criterion.

Since the TBM tracking depends on the processing of the feature PIR signals and the weighted mean center scheme, observe that for Routes 01 and 03, the dominant source of estimation error is from the zigzag estimates, where the average RMSE relationship between *x* and *y* is approximately linear with slopes of 0.83 and 1, respectively. For Route 02, similar to the Kalman filter tracking, the dominant source of estimation error is from the *x*-direction movement, which leads to a smaller estimation error. For Routes 04 and 05, the sources of estimation error are from the zigzag estimates and direction changes.

Based on the tracking performance, we compare the proposed Kalman filter and TBM tracking schemes from two perspectives. From an input information perspective, to improve the localization accuracy and describe the tendency of a moving target, the central coordination of Zone(z) was applied as the input for the Kalman filter to obtain a full target trajectory. In contrast, for the TBM, the state of each PIR sensor $s_{k}$, corresponding to the zones regarding the target movement, was used to provide the information about the regions traversed.

From a signal processing perspective, consider the tracking ability. Referring to Section 5.3.1, the Kalman filter scheme provides a full trajectory. Similarly, for the TBM scheme with the feature PIR signals and the weighted mean center scheme, the probability variation of the target being in each zone and a set of inputs were utilized to generate a processed target trajectory. For Routes 01–03 with the same moving direction, although the TBM scheme has a larger estimation error, it can also reveal certain information about the target trajectory. For Routes 04–05 with direction changes, even with relatively slight deviations from the trajectory, the TBM scheme properly responds to these sharp turns.

#### 5.3.3. TBM-Based Hybrid Approach

This set of experiments assessed the improvement in the estimation accuracy achieved by the hybrid system consisting of parts A, B, C, and D, in Figure 5. Referring to Table 4, Table 5 and Table 6, the average RMSEs of Routes 01~05 with the proposed hybrid system all showed improvements.

**Figure 10:**
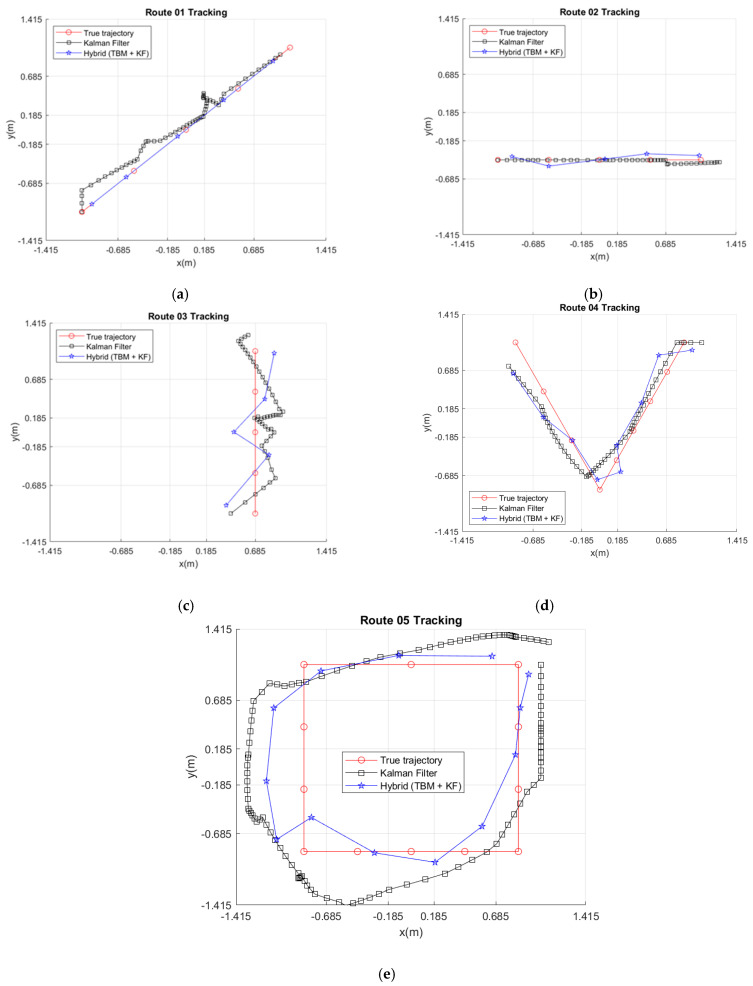
Typical examples of tracking results with the hybrid approach and Kalman filter: (**a**) Route 01, (**b**) Route 02, (**c**) Route 03, (**d**) Route 04, and (**e**) Route 05.

For Route 01, the confusing PIR signals may lead to deviations in the central zone coordinates for the inputs of the Kalman filter. In contrast, the hybrid approach utilizes the probability variation of the target being in each zone and the weighted mean center scheme to calibrate the input to the Kalman filter. Consequently, as shown in Figure 10a, the deviation of the estimated trajectory was suppressed. Compared with the Kalman filter and the TBM approaches, the improvement percentages of the average RMSE of the hybrid approach were about 9.7% and 43.3%, respectively.

For Route 02, Table 2 shows that the TBM input reflects the signal status of the sensing zones, which specifies possible target areas. Figure 10b shows a mild deviation of the experimental trajectory with the Kalman filter approach, where the hybrid approach relatively precisely follows the trajectory. Compared with the Kalman filter and the TBM approaches, the hybrid approach generates a better tracking trajectory, where the improvement percentages of average RMSE of the proposed system with the hybrid approach were about 28.8% and 42.1%, respectively.

For Route 03 (i.e., walking along the boundary line between two adjacent zones), once again, Figure 10c shows that the hybrid approach effectively deals with the confusing PIR signals via signal preprocessing and controls the trajectory more precisely. Comparing with the Kalman filter and the TBM approaches, the improvement percentages of the average RMSE of the hybrid approach were about 6.2% and 26.7%, respectively.

For Route 04, considering diagonal routes with the change of direction, Table 6 shows that the dominant source of estimation error is from the y-direction movement. Comparing with the Kalman filter and the TBM approaches, the improvement percentages of the average RMSE of the hybrid approach were about 4.4% and 16.1%, respectively. Although the hybrid approach only shows a slight improvement in RMSE performance over that of the Kalman filter, Figure 10d shows that the hybrid approach has better tracking fidelity.

For Route 05, walking along a square route, Table 6 shows that the average RMSE relationship between *x* and *y* is approximately linear with a slope of 1. Figure 10e shows that the hybrid approach benefits from the quick response of the TBM scheme to the route characteristics. Comparing with the Kalman filter and the TBM approaches, the improvement percentages of the average RMSE of the hybrid approach were about 100.3% and 53.3%, respectively, which shows that the hybrid approach is especially promising for tracking with direction changes.

#### 5.3.4. Discussion

Wei [26] emphasized that response speed is an important metric for real-time tracking applications. Considering the sampling accuracy and the hardware processing ability, the sampling frequency of the PIR sensor was chosen as 75 Hz. Based on the system operations, tracking with the Kalman filter approach was responded to about every 0.24 s, while tracking with the TBM approach was performed with a lower response speed (about every 0.75 s). Referring to the delay requirement (<1 s) in [27], the response speed of the proposed system is acceptable. Table 7 summarizes the maximum allowable moving speeds of routes 01~05, which are about 0.4 m/s and 0.7 m/s for the boundary and non-boundary routes, respectively. Thus, the proposed system demonstrated good detection capability and a precise description of trajectory tendency such that the performance balance between estimation accuracy and detection capability can be achieved.

## 6. Conclusions

This paper proposed a non-wearable system for cooperative indoor human localization. We conducted three experiments to assess the system performance, integrating the PIR detector design and the signal processing for improving PIR signal quality. Based on the feature PIR signals, Kalman filter, TBM, and TBM-based hybrid approaches were further used to verify and refine the system performance. In Experiment 2, the proposed system with TBM clearly indicated the sequence of zones passed by the target, which provides clear clues to the TBM about the possible range of the target and the target trajectory. In Experiment 3, a Kalman filter was applied as a quantitative benchmark to show the performance improvement. For a TBM tracking scheme, this paper integrates the quantified beliefs and the weighted mean center scheme with the sensor deployment and the status of sensing zones simultaneously, which not only qualitatively provides the information about the regions traversed, but also quantitatively estimates human movements. Furthermore, this study further developed a TBM-based hybrid approach, integrating a belief-driven approach (TBM) and a model-based approach (Kalman filter), to improve the tracking performance. Given the detection block sizes quite close to the target size, the proposed system performed well under these experimental scenarios with acceptable response speeds. Based on the proposed system, a multi-tier computing and communication architecture [28] may be feasible based on the proposed PIR detectors, relay gateways (e.g., device local controllers), and a base station (e.g., a server platform), which provides a hierarchical indoor localization framework for PIR sensor networks. Accordingly, this application architecture can collect and process the sensing data, relay the position information, and illustrate the position on a map.

Although the proposed system is promising, there remain certain limitations with the proposed detection system. The current digital signals do not contain enough information to identify or distinguish people. In the future, we plan to thoroughly explore the analog outputs from the PIR sensors (e.g., amplitude, phase, and frequency) to further characterize the features of the subjects [29]. Such characterization might also help to distinguish the targets from other heat sources (e.g., pets) [30]. Adaptive thresholding of the PIR analog signals may help to reduce such environmental disturbances. Furthermore, we plan to design a robust indoor human localization system for multiple targets, which may include improving the PIR detector design, reshaping the detection area of the PIR detector (or system), exploring more efficient signal processing algorithms, and deriving effective and reliable localization algorithms.

## Figures and Tables

**Table 1:** Summary of the PIR Detection Systems.

Works	Sensor Location	Processing Techniques	Observation Space	Average RMSE (m)
[1] with Kalman Filter	Ceiling	Short-Time Energy, Spatial Segmentation	5 m × 5 m	0.508
[15] with Kalman Filter	Ceiling	Spatial Segmentation	5 m × 5 m	0.68
Naive Bayes Method [13]	Floor	Training/Classifier, Spatial Segmentation	10 m × 10 m	0.49
Credit-Based Method [14]	Floor	Crossing Location	10 m × 10 m	0.384
Sensor Selection and Calibration Method [20]	Wall	Probability Model-based Calibration	6 m × 6 m	0.35
The Proposed System with Kalman Filter	Ceiling	Feature Signal Extraction	5 m × 5 m	0.254
The Proposed System with TBM-based Hybrid Method	Ceiling	Feature Signal Extraction	5 m × 5 m	0.219

**Table 2:** The Inputs of TBM.

Route 01* Zone(1→7→13→19→25)							
	$s_{k}$	*s*_1_	*s*_2_	$s_{3}$	*s*_4_	*s*_5_	$s_{6}$
Input *i*							
Input 01		cov_1_	cov_7_	${\mathrm{cov}}_{13}$	${\bar{\mathrm{cov}}}_{18}$	cov_25_	X
Input 02		cov_1_	cov_7_	cov_13_	${\bar{\mathrm{cov}}}_{18}$	${\underline{\mathrm{cov}}}_{19}$	${\underline{\mathrm{cov}}}_{25}$
Input 03		cov_1_	cov_7_	cov_13_	cov_19_	cov_25_	X
Route 02 Zone(16→17→18→19→20)							
cov_16_		cov_17_	cov_18_	cov_19_	cov_20_	X	
Route 03 Zone(4,5→9,10→14,15→19,20→24,25)							
cov_5_		${\underline{\mathrm{cov}}}_{15}$	${\underline{\mathrm{cov}}}_{25}$	X	X	X	
cov_5_		${\underline{\mathrm{cov}}}_{15}$	${\underline{\mathrm{cov}}}_{14}$	${\underline{\mathrm{cov}}}_{25}$	X	X	

**Table 3:** The highest three $\Delta Prob_{k-1∼k}^{z}$ values of Input 01 for Route 01*.

(k−1)∼kth Sampling	0∼1	1∼2	2∼3	3∼4	4∼5
first	$\Delta Prob_{0∼1}^{1}$ (0.4569)	$\Delta Prob_{1∼2}^{7}$ (0.2376)	$\Delta Prob_{2∼3}^{13}$ (0.1937)	${\bar{\Delta Prob}}_{3∼4}^{18}$ (0.2049)	$\Delta Prob_{4∼5}^{25}$ (0.3939)
second	$\Delta Prob_{0∼1}^{2}$ (0.0517)	$\Delta Prob_{1∼2}^{12}$ (0.0837)	$\Delta Prob_{2∼3}^{18}$ (0.0393)	$\Delta Prob_{3∼4}^{19}$ (0.0946)	$\Delta Prob_{4∼5}^{24}$ (0.0904)
third	$\Delta Prob_{0∼1}^{6}$ (0.0517)	$\Delta Prob_{1∼2}^{8}$ (0.0837)	$\Delta Prob_{2∼3}^{14}$ (0.0393)	$\Delta Prob_{3∼4}^{17}$ (0.0556)	$\Delta Prob_{4∼5}^{20}$ (0.0528)

**Table 4:** Kalman Filter: The average RMSE of Routes 01~05.

Experimental Route	Avg. RMSE*_x_*	Avg. RMSE*_y_*	Avg. RMSE (m)
Route 01	0.157	0.148	0.216
Route 02	0.207	0.080	0.222
Route 03	0.185	0.266	0.324
Route 04	0.199	0.205	0.286
Route 05	0.551	0.372	0.665

**Table 5:** TBM: The average RMSE of Routes 01~05.

Experimental Route	Avg. RMSE*_x_*	Avg. RMSE*_y_*	Avg. RMSE (m)
Route 01	0.219	0.265	0.344
Route 02	0.266	0.060	0.273
Route 03	0.292	0.295	0.415
Route 04	0.152	0.279	0.318
Route 05	0.384	0.334	0.509

**Table 6:** TBM-based Hybrid Approach: The Average RMSE of Routes 01~05.

Experimental Route	Avg. RMSE*_x_*	Avg. RMSE*_y_*	Avg. RMSE (m)
Route 01	0.126	0.149	0.195
Route 02	0.141	0.072	0.158
Route 03	0.232	0.196	0.304
Route 04	0.111	0.251	0.274
Route 05	0.232	0.238	0.332

**Table 7:** The Maximum Allowable Moving Speed (m/sec) of Routes 01~05.

Experimental Route	Kalman Filter	TBM	Hybrid
Route 01	0.883	0.823	0.743
Route 02	1.053	0.673	0.712
Route 03	0.712	0.471	0.410
Route 04	0.802	0.727	0.703
Route 05	0.884	0.724	0.766

## Data Availability

The author at the Wireless and Communication Networking Laboratory, located at the Department of Electrical Engineering, National Chung Hsing University, Taiwan, was the subject of the experiments. The author consented to participate in this research study.
